# Spatiotemporal image quality of virtual reality head mounted displays

**DOI:** 10.1038/s41598-022-24345-9

**Published:** 2022-11-24

**Authors:** Chumin Zhao, Andrea S. Kim, Ryan Beams, Aldo Badano

**Affiliations:** Center for Devices and Radiological Health, U.S. Food and Drug Administration, 10903 New Hampshire Ave, Silver Spring, MD 20993 USA

**Keywords:** Imaging techniques, Medical imaging, Electronics, photonics and device physics, Optical physics

## Abstract

Virtual reality (VR) head mounted displays (HMDs) require both high spatial resolution and fast temporal response. However, methods to quantify the VR image quality in the spatiotemporal domain when motion exists are not yet standardized. In this study, we characterize the spatiotemporal capabilities of three VR devices: the HTC VIVE, VIVE Pro, and VIVE Pro 2 during smooth pursuit. A spatiotemporal model for VR HMDs is presented using measured spatial and temporal characteristics. Among the three evaluated headsets, the VIVE Pro 2 improves the display temporal performance using a fast 120 Hz refresh rate and pulsed emission with a small duty cycle of 5%. In combination with a high pixel resolution beyond 2 k $\times$ 2 k per eye, the VIVE Pro 2 achieves an improved spatiotemporal performance compared to the VIVE and VIVE Pro in the high spatial frequency range above 8 cycles per degree during smooth pursuit. The result demonstrates that reducing the display emission duty cycle to less than 20% is beneficial to mitigate motion blur in VR HMDs. Frame rate reduction (e.g., to below 60 Hz) of the input signal compared to the display refresh rate of 120 Hz yields replicated shadow images that can affect the image quality under motion. This work supports the regulatory science research efforts in development of testing methods to characterize the spatiotemporal performance of VR devices for medical use.

## Introduction

Virtual reality (VR) simulates a real-time immersive experience using a head mounted display (HMD) enabling numerous new opportunities in entertainment^1^, communication^2^, education^3^, manufacturing^4^, and military^5^ applications. As an emerging field, VR applications in medical and healthcare involve 3D visualization of patient anatomical imaging^6,7^, surgical planning^8,9^, diagnostics^10^, therapy^11–13^, pain management^14,15^, and medical training^16,17^. Image quality in VR HMDs has been improved over the past decades thanks to the rapid advancement in display technologies and software. However, spatial and temporal image artifacts remain visible, degrading image quality and raising concerns in the safety and effectiveness for VR medical use.

In the spatial domain, the screen door effect (SDE) arises in VR displays when the virtual image and individual pixels are magnified^18,19^. The SDE is particularly common in headsets implementing the PenTile organic light emitting diode (OLED) displays. The PenTile matrix uses a diamond-shaped RGBG sub-pixel layout that doubles the number of green sub-pixels^20^. However, pixel resolution and fill factor are insufficient after magnification, creating pixelation and SDE that degrades the spatial image quality. Fast-switching liquid crystal display (LCD)^21,22^ is an alternative display technology that uses a high definition (e.g., greater than 2 k $\times$ 2 k pixels per eye) RGB panel to mitigate the SDE. One drawback of the fast-switching LCDs is the limited dynamic range compared to the OLED displays. Regardless of the display technologies, the spatial resolution of VR HMDs degrades at the periphery of the display field of view (FOV) resulting in a blurry image with chromatic aberration at wide viewing angles^23,24^.

An immersive VR environment requires not only a high spatial resolution but also a fast device temporal response to minimize temporal image artifacts. Continuous efforts to improve the display refresh rate^25^ help reduce the temporal artifacts such as flicker, motion blur, judder and multiple images^26,27^. Modern VR HMDs are typically driven at a refresh rate higher than 60 Hz to reduce flickering and motion-to-photon latency^28,29^. Mitigation of motion blur can include short-pulse emissions in combination with a high display refresh rate (higher than 90 Hz) in advanced VR displays using both OLED^30^ and LCD technologies^21,31^. The short pulses are generated by the OLED emission cycles on the pixel level, while by fast switching the backlight of the LCDs. In addition to the display refresh rate, the system motion-to-photon latency during sensing, tracking, and rendering process may also contribute to temporal artifacts such as judder and multiple images^27,28^.

To study the spatiotemporal performance of VR HMDs with a moving object in the scene, gaze tracking needs to be considered^32,33^. During smooth pursuits, human eyes track the moving target at the same velocity^25,34^. As the display image is static within a frame, the relative perceived motion with respect to the fixed image results in motion blur that is highly associated with the temporal response of the display^26^. It is worth mentioning that a human eye response latency of approximately 100 ms is required for the eye to realize a smooth pursuit^35^. For very fast motion at speeds greater than 40°/s, the eye movements switch to saccades to catch the fast stimulus by an instant motion of the gaze as fast as 180°/s^36^. Masaoka presented a simulation framework to obtain the spatiotemporal resolution of pixelated displays during smooth-pursuit eye movement^32^. The present study extends the previous work to VR HMDs with pulsed emission and validates the results using empirical spatial, temporal and spatiotemporal measurements. The impact of spatial and temporal performance on the spatiotemporal characteristics is investigated. Finally, visualizations and image quality with motion artifacts are simulated and quantified.

## Methods

### VR head mounted displays

Spatiotemporal responses of three VR headsets were characterized in this study: the HTC VIVE, VIVE Pro, and VIVE Pro 2 (HTC Corporation, Taiwan). Both the VIVE and VIVE Pro implement the PenTile OLED backplanes with a display refresh rate up to 90 Hz and a horizontal field of view (FOV) of approximately 110$^{\circ }$. The VIVE Pro improves the pixel resolution to 1440 $\times$ 1600 per eye compared to the VIVE with 1080 $\times$ 1200 pixels per eye.

The latest generation VIVE Pro 2 switches to a fast (up to 120 Hz) high-resolution dual RGB low persistence liquid crystal display (LCD) panel with a 2448 $\times$ 2448 pixel resolution per eye and a slightly wider horizontal FOV of approximately 120$^{\circ }$.

The VIVE, VIVE Pro, and VIVE Pro 2 HMDs were set to the maximum brightness levels of 208, 130 and 79 cd/m$^2$ respectively during the measurements. The luminance was measured using an ILT5000 research radiometer (International Light Technologies, USA).

**Figure 1:**
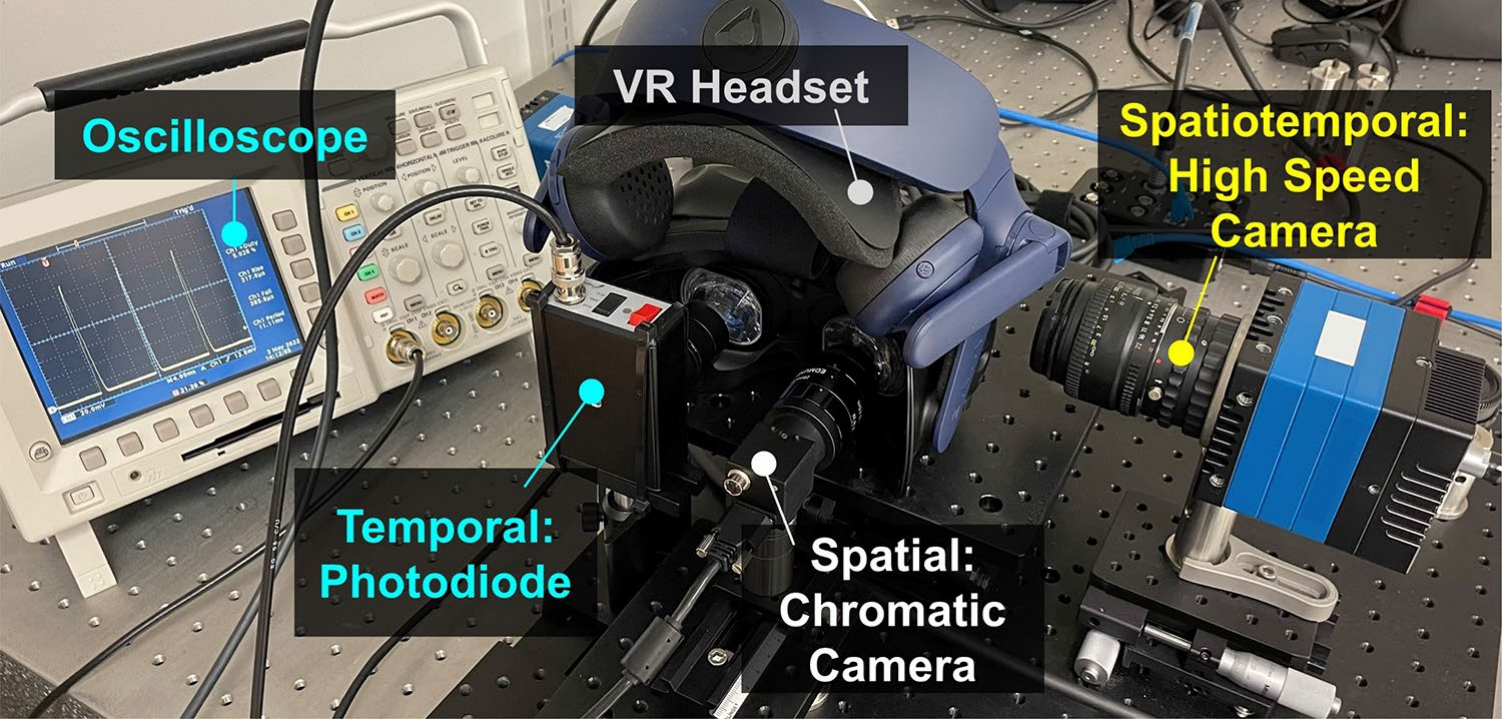
Experimental bench setup of spatial, temporal, and spatiotemporal measurements for VR HMDs.

**Figure 2:**
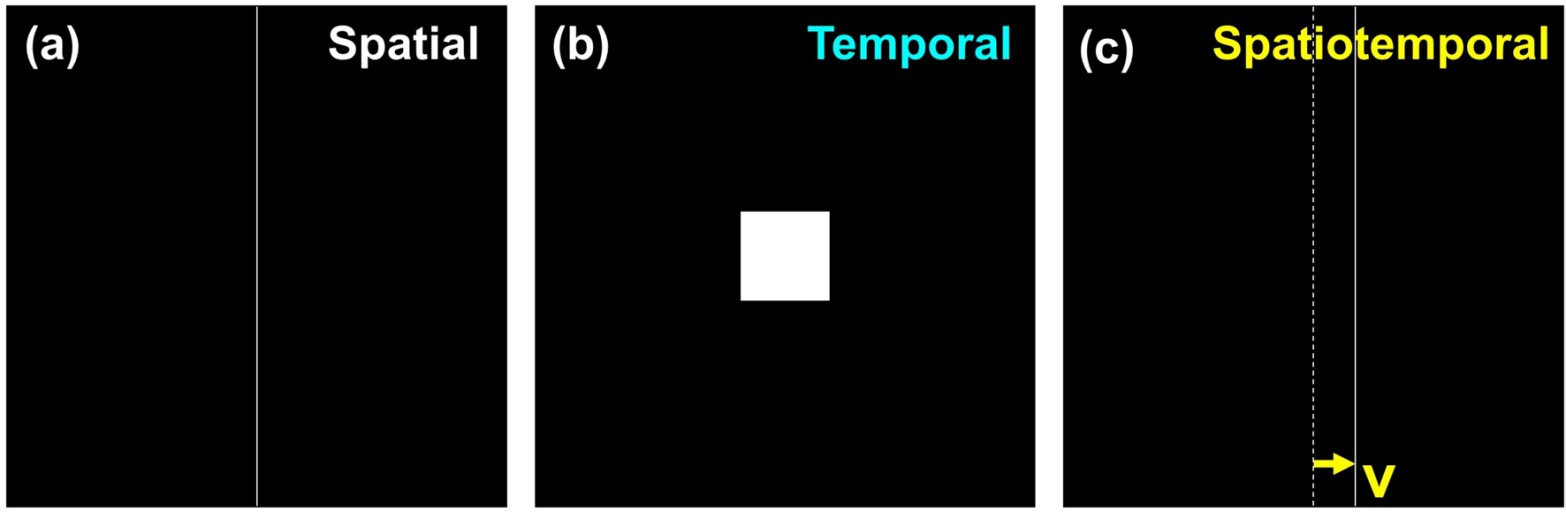
Illustrations of the test patterns used for the spatial (**a**)—a static single pixel line, temporal (**b**)—a 100 $\times$ 100 pixels white box, and spatiotemporal measurements (**c**)—a moving line with a velocity *v* in degree per second.

### Spatial resolution measurement

Figure 1 shows the experimental setup to measure the spatial resolution of VR HMDs. The evaluated VR HMD was mounted on an optical bench. A static single-pixel vertical line (Fig. 2a) was placed at the the center of the right-eye FOV. The tested VR HMD was operated as an external display driven by an Nvidia GeForce RTX 2080 Ti graphic card (Nvidia, USA). Therefore, no software-based spatial or motion smoothing was applied during the measurements, and system latency was excluded from this study. A chromatic CMOS camera (FLIR Blackfly BFS-U3-122S6C-C, Teledyne FLIR, USA) with a 25 mm focal length lens (f/2.8) was aligned with the static line. The camera was placed at about 25 mm from the lens of the HMD and centered by displaying multiple lines with 60-pixel separation across the display FOV. The camera position and focus were determined when the sharpest line was resolved with visible sub-pixel patterns at the center of the display FOV. At the same time, symmetric aberration and luminance between lines located on the left and right half of the image were achieved. The exposure time of the FLIR camera was 33.3 ms for spatial resolution measurements. The camera gain was adjusted according to display luminance to maximize the obtained signal without saturation. The FLIR camera achieves a high pixel resolution of 4096 $\times$ 3000 with the angular resolution calibrated as $7.8\times 10^{-3}$ degree per pixel (approximately 0.47 arcmin per pixel). The angular resolution of the chromatic camera is greater than that of the VR displays (greater than 1.8 arcmin per pixel) providing sufficient spatial sampling.

Note that the spatial resolution of VR HMDs is sensitive to the viewing angle and displacement of the eye or camera within the eyebox^23,37^. The spatially dependent spatial resolution of VR HMDs has been investigated in our previous work^24^ and we observed substantial spatial resolution degradation at the periphery of the VR display FOV. The results presented in this study only shows the optimal spatial and spatiotemporal capabilities of the VR HMDs at the center of the display FOVs.

The static line spread function ($LSF_s$) of a single-pixel vertical line was obtained by averaging over 100 rows to minimize the image noise and to average over the subpixels. The static modulation transfer function ($MTF_s$) was subsequently computed as the normalized fast Fourier transform (FFT) of the $LSF_s$.

### Temporal response measurement

A silicon photodetector (Model 1621, Newport, USA) in combination with an oscilloscope was used to characterize the temporal performance of each HMD (Fig. 1). The photodiode is sensitive to visible light wavelengths ranging from 350 to 1000 mm with a fast nanosecond response time. As shown in Fig. 2b, a white box of 100 $\times$ 100 pixels was centered within the display FOV to minimize the temporal averaging over scanned rows. The temporal waveform, period, refresh rate, duty cycle (defined as the ratio of display emission time and period), rise and fall times were obtained from the oscilloscope measurements and compared between the three evaluated VR HMDs.

### Spatiotemporal characteristics for smooth-pursuit eye movement

Prior to the spatiotemporal measurements, the temporal waveform of a flickering 1-on-1-off video of the white box was captured using the photodiode method to validate the display refresh rate (i.e., no frame rate reduction during the spatiotemporal measurements).

**Figure 3:**
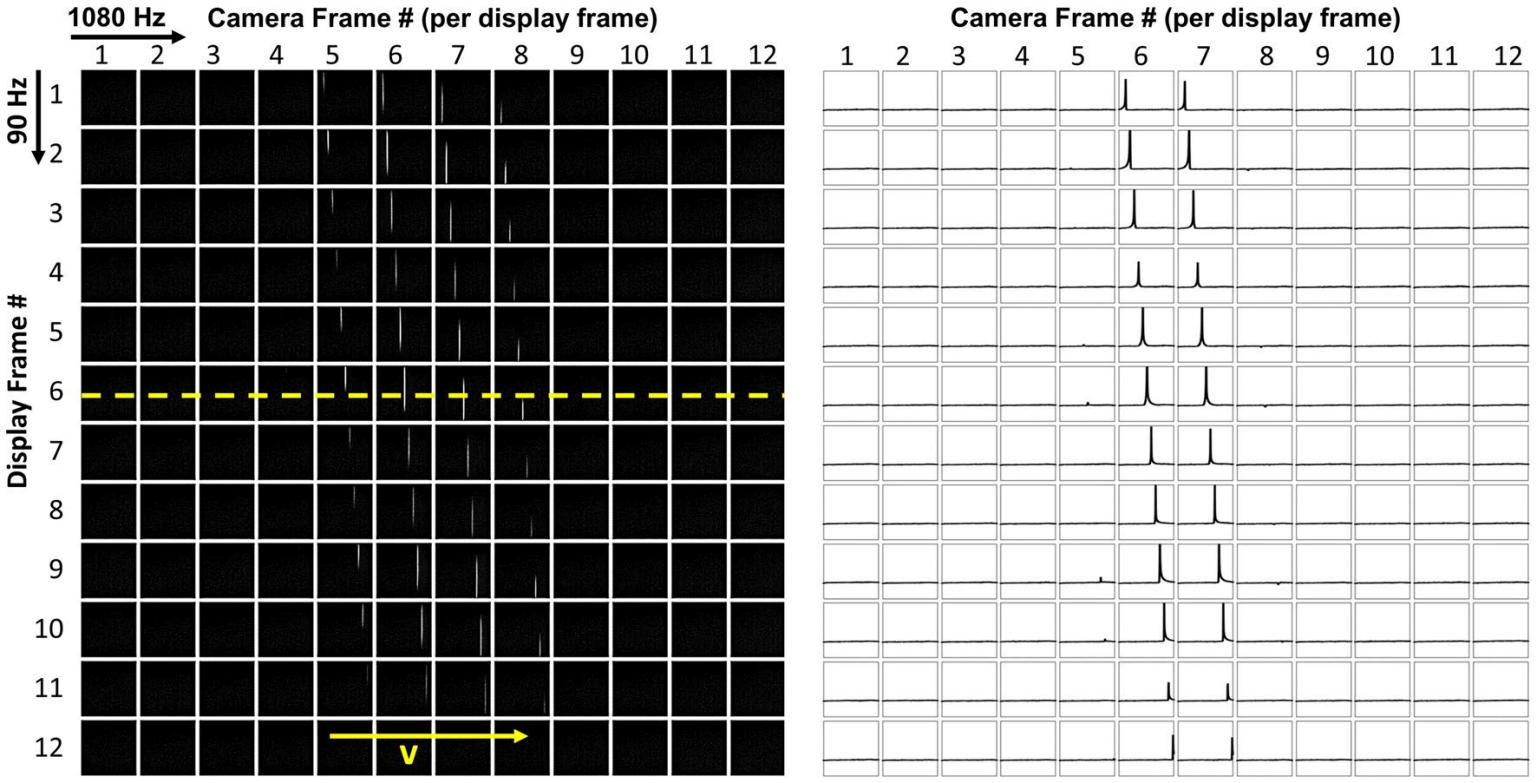
Illustration of the spatiotemporal measurements and the LSFs of a moving line shown on the 90 Hz VIVE Pro using the high-speed camera at 1080 Hz.

To characterize the spatiotemporal performance of VR HMDs, a high-speed camera (pco.dimax cs4, PCO, Germany) with a frame rate up to 1102 Hz and a resolution of 2016 $\times$ 2016 was used to capture multiple images per display frame. A 50 mm lens with an f/2.8 aperture was used. The camera frame rate was set to 1080 Hz (exposure time of about 0.9 ms). As illustrated in Figs. 2c and 3, for spatiotemporal measurements, an uncompressed moving line video with a speed *v* in degree per second was played on the VR headset, while the high-speed camera captured *N* images per display frame. For example, for the VIVE and VIVE Pro with a 90 Hz refresh rate, *N* is the division of camera frame rate over the VR display refresh rate, i.e., $N = 1080/90 = 12$.

**Figure 4:**
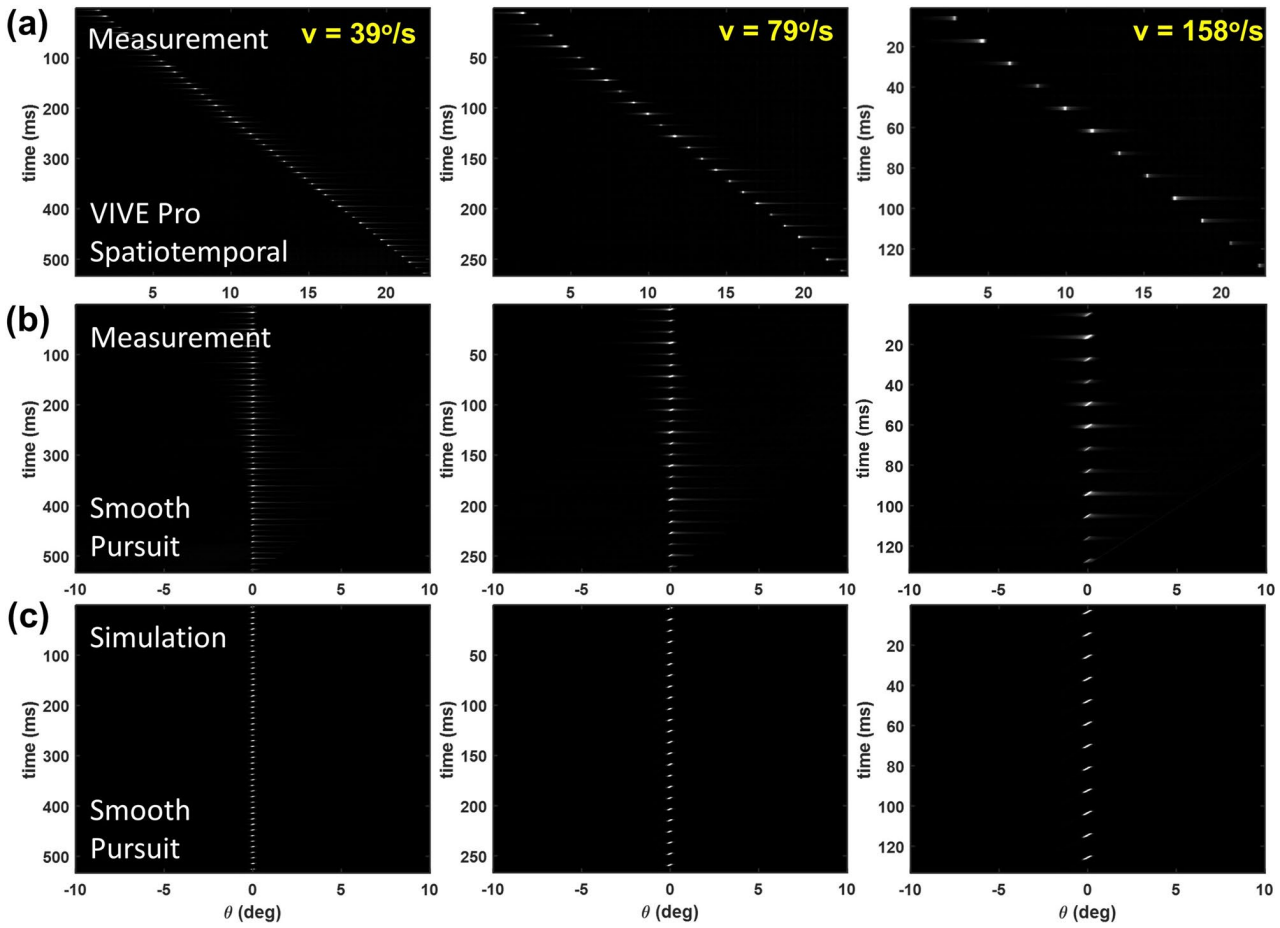
(**a**) Measurements of the time-dependent LSFs with respect to a static eye at target motion velocity of 39 (first column), 79 (second column), and 158°/s (third column) using the VIVE Pro. (**b**) Same measurements as in (**a**) assuming a smooth-pursuit eye movement. (**c**) Simulated time-dependent LSFs with the same target velocity as in (**a**) during smooth pursuit.

The LSF of the moving line in each captured image was obtained by averaging over 100 central rows (see the right half of Fig. 3). Note that the white line is static within each display frame, while laterally shifted by *v*/*f* degrees between neighboring display frames, where *f* is the display refresh rate. Also shown in Fig. 3, within each display frame of the VIVE Pro, the emission is only on for a short period of time (approximately 2 frames/12 frames $\approx$ 17%).

Figure 4a illustrates the propagation of the LSFs over time (*t*) at various motion speeds: 39, 79 and 158°/s corresponding to motion speeds of 8, 16 and 32 pixel per frame of the single-pixel line on the VIVE Pro. In each display frame of 11.1 ms, 12 LSFs are captured with an emission time of approximately 1.85 ms (corresponding to two LSFs as shown in Fig. 3). Mathematically, with respect to a static eye, a moving scene at time *t* and frame *i* ($I_i$) can be described as

1 $$\begin{aligned} {\left\{ \begin{array}{ll} I_i(x,y,t) = w(t) I_s(x,y), \\ I_{i+1}(x,y,t_{i+1}) = I_i(x - v/f,y, t_{i}), \\ \end{array}\right. } \end{aligned}$$

where $I_s(x,y)$ is the static image, *w*(*t*) is the normalized temporal weights representing the temporal waveform of the VR HMD, and $t_i$ is the starting time of frame *i*.

Next, we assume the smooth-pursuit eye movement, i.e., the human eye perfectly tracks the moving target at the same constant speed *v*. Note that this assumption is only valid for motion speed up to approximately 40°/s without triggering the saccadic eye movement^38^. The higher motion speeds are only used for proof of concept and validation of the spatiotemporal response model in the following section. The latency of smooth pursuit response of approximately 100 ms at the beginning of the tracking process^35^ is also ignored.

During a smooth-pursuit event, the human eyes are relatively static to the moving target. However, within each display frame, the display content is not updated (i.e., the display image is static), while the gaze keeps moving resulting in a relative motion of the display image at $v' = -v$ with respect to the moving eye.

As illustrated in Fig. 4b, a moving scene shown on a VR HMD at time *t* ($I(x,y,t)'$) with respect to a tracking eye in smooth-pursuit motion is given by

2 $$\begin{aligned} {\left\{ \begin{array}{ll} I_i(x,y,t)' = w(t)\cdot I_s(x + v(t-t_i),y), \\ I_{i+1}(x,y,t_{i+1})' = I_i(x,y,t_i)'. \\ \end{array}\right. } \end{aligned}$$

In this case, $I(x,y,t)'$ is static between display frames, while moving laterally within a display frame. Prior to the lateral shift, we interpolated the time-dependent LSFs in the time domain of Fig. 4 to realize sufficient temporal sampling.

### Spatiotemporal response model

Measurements of the spatiotemporal responses of VR HMDs require a high-speed camera with high spatial resolution at the same time. The measurement imaging system should also have sufficient optical performance to not affect the measured results on resolution or image quality. Alternatively, in this section, we present a simple spatiotemporal model that simulates the dynamic LSFs and MTFs using only the measured spatial and temporal characteristics.

Specifically, the spatial resolution and temporal response measurements provide the static LSF ($LSF_s(x)$) and display luminance waveform (*w*(*t*)), respectively. Based on Eq. (2), the time-dependent LSF at time *t* and frame *i* under smooth pursuit can be simulated by

3 $$\begin{aligned} {\left\{ \begin{array}{ll} LSF_i(x,t)' = w(t)\cdot LSF_s(x + v(t-t_i)), \\ LSF_{i+1}(x,t_{i+1})' = LSF_i(x,t_i)'. \\ \end{array}\right. } \end{aligned}$$

Figure 4c shows an example of the simulated time-dependent LSFs using the VIVE Pro compared to the measured results in (b) during smooth pursuit tracking. We observe a wider lateral shift within a display frame by increasing the target velocity that is consistent with the measured results.

To evaluate the impact of display temporal characteristics on spatiotemporal image quality, we can also vary the temporal weights *w*(*t*) using a rise ($\tau _r$) and fall time constants ($\tau _f$) that can be derived from the rise and fall times of the measured waveforms. Therefore, the temporal weights can be expressed as

4 $$w(t) = \left\{ {\begin{array}{*{20}l} {1 - e^{{ - t/\tau _{r} }} ,\qquad \qquad \qquad \qquad \quad } \hfill & {t \le D/f} \hfill \\ {[1 - e^{{ - D/\tau _{r} f}} ] \cdot e^{{ - (t - D/f)/\tau _{f} }} ,\qquad } \hfill & {t > D/f} \hfill \\ \end{array} } \right.$$

where *D* is the duty cycle of the emission and *f* is the refresh rate.

The dynamic LSF ($LSF_d(x)$) with smooth-pursuit eye movement was obtained by integrating the $LSF(x,t)'$ over time *t*. The dynamic MTF ($MTF_d$) was subsequently computed as the FFT of $LSF_d$, i.e.,

5 $$\begin{aligned} MTF_d(f_x) = Norm\{FFT[LSF_d(x)]\} = Norm\{FFT[\int _t LSF(x,t)' dt]\}. \end{aligned}$$

**Figure 5:**
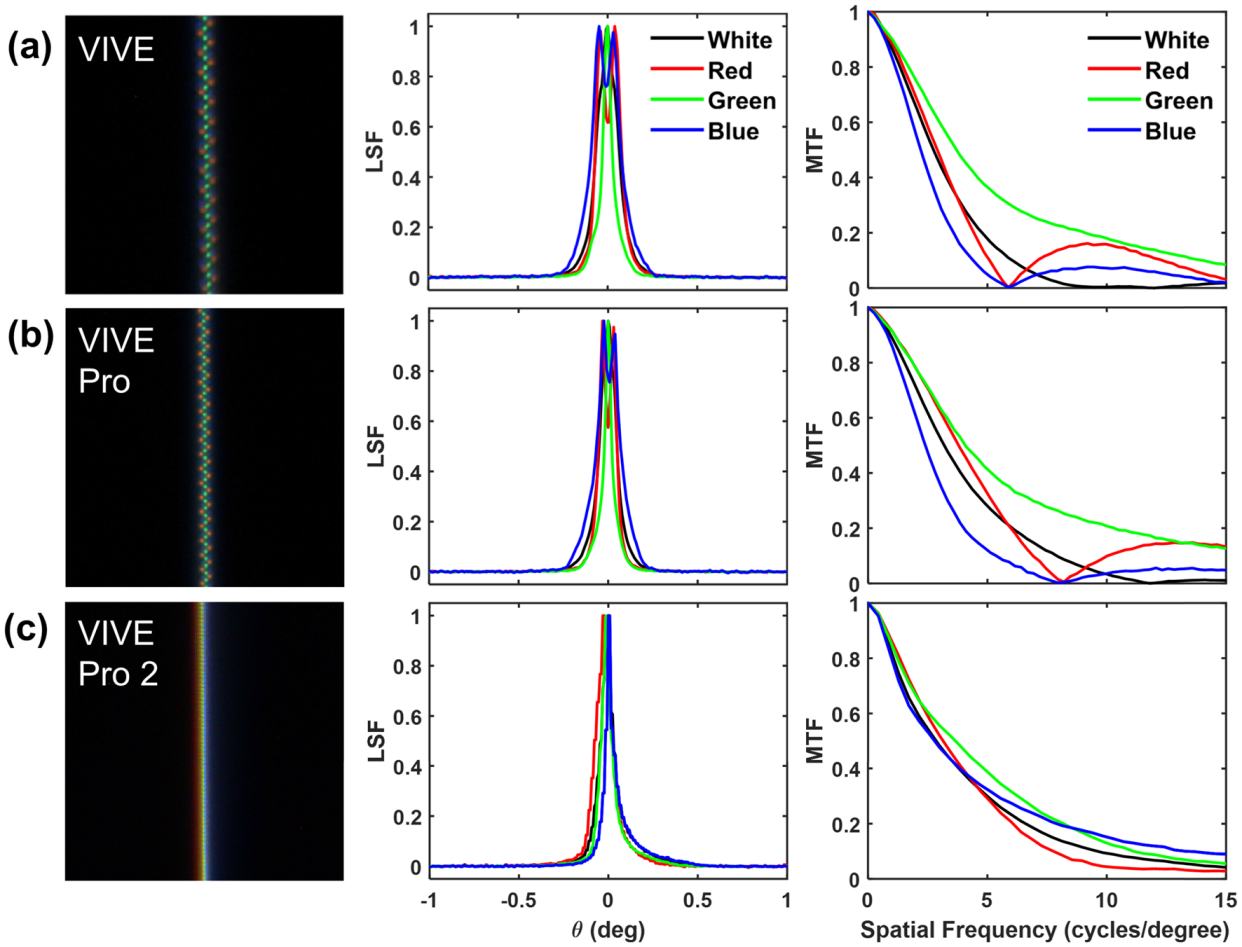
Single pixel white line images (first column), LSFs (second column) and MTFs (third column) of white, red, green and blue vertical lines for the VIVE (**a**), VIVE Pro (**b**), and VIVE Pro 2 (**c**).

## Results

### Spatial performance of VR displays

Figure 5 compares the spatial resolution of the VIVE (a), VIVE Pro (b) and VIVE Pro 2 (c). As illustrated in the example images of the static single-pixel white lines captured by the FLIR camera, the VIVE and VIVE Pro present a zigzagging pattern of the straight line using the PenTile OLED subpixel layout with approximately doubled spatial resolution of the green subpixels compared to the red and blue as shown in the LSFs and MTFs. The cutoff frequencies of the red and blue lines for the VIVE and VIVE Pro are approximately 6 and 8 cycles/$^{\circ }$, respectively. The white color yields slightly improved spatial resolution of approximately 9 and 12 cycles/$^{\circ }$ for the VIVE and VIVE Pro by averaging the luminance of primary color subpixels.

The VIVE Pro 2 implements a RGB LCD backplane with a 2448 $\times$ 2448 pixel resolution per eye. However, the measured spatial resolution is not drastically improved compared to the VIVE or VIVE Pro with lower pixel resolution. Although the high pixel resolution of the VIVE Pro 2 improves the high frequency (e.g., greater than 10 cycles/$^{\circ }$) MTF of white pixels, the low-frequency performance (less than 5 cycles/$^{\circ }$) is limited. We suspect that the drop in low-frequency performance in VIVE Pro 2 is primarily due to the difference in display technology and architecture. Specifically, the LCD technology in the VIVE Pro 2 implements stacked liquid crystal modules including polarizers and color filters, which are not present in OLED displays such as the VIVE and VIVE Pro. The additional optical components and layers in LCD displays may result in additional image blur that affects image resolution in the low-frequency range. One benefit of the LCD compared to the PenTile OLED is the increased pixel fill factor, i.e., larger emission area per pixel. Therefore, the LCD panel of the VIVE Pro 2 provides a smoother visualization of the virtual image without the SDE shown on the VIVE and VIVE Pro.

### Temporal response

**Figure 6:**
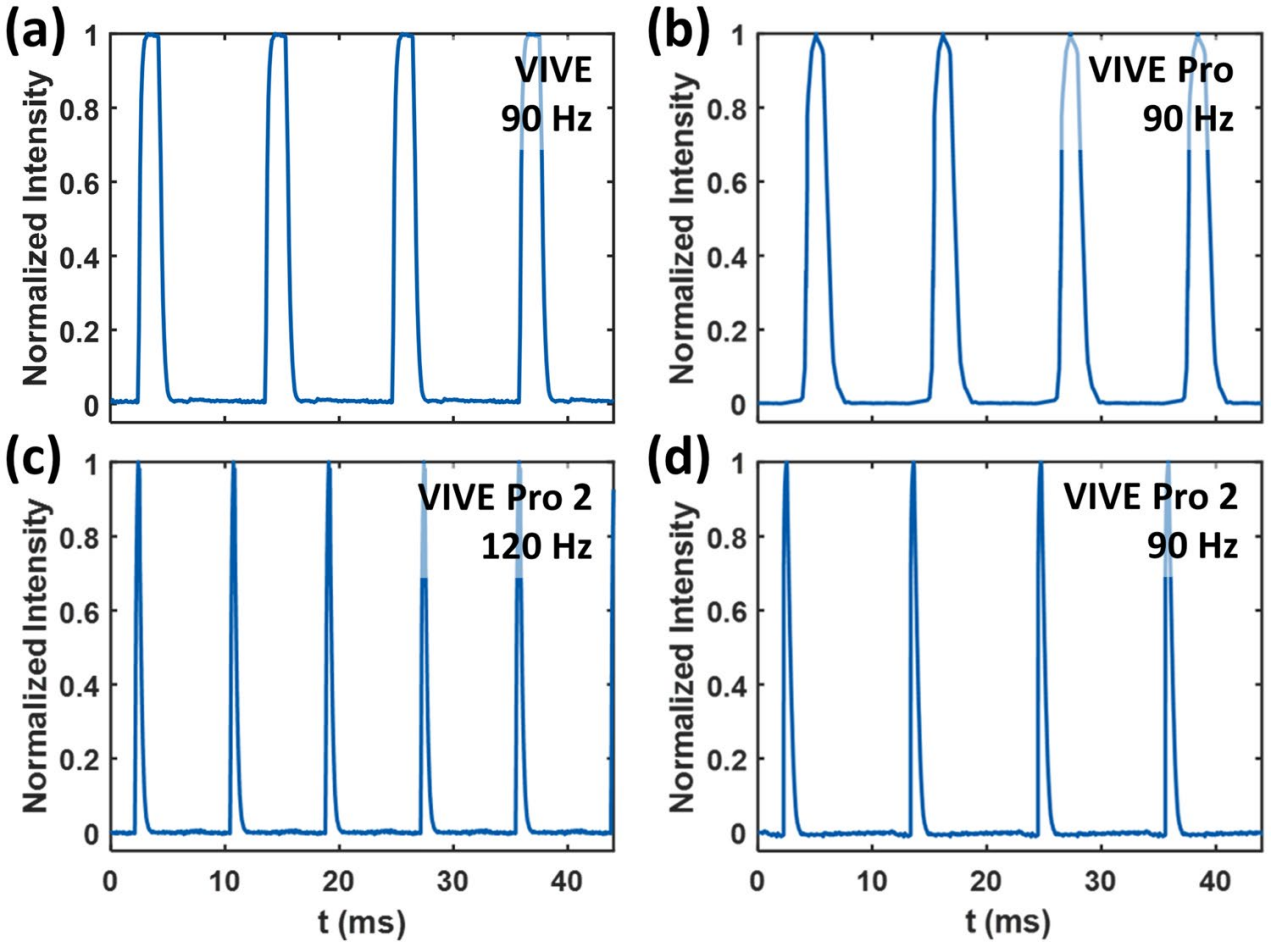
Temporal responses of (**a**) the VIVE at 90 Hz, (**b**) the VIVE Pro at 90 Hz, (**c**) the VIVE Pro 2 at 120 Hz, and (**d**) the VIVE Pro 2 at 90 Hz.

As shown in Fig. 6, pulsed emission is used for all evaluated VR HMDs, which advances from the conventional flat-panel displays. The photodiode measurements demonstrate that the VIVE and VIVE Pro can operate at up to 90 Hz, while the refresh rate of the VIVE Pro 2 can be further upgraded to 120 Hz. The duty cycle of the VIVE and VIVE Pro is 17% by modulating the OLED emission time. The rise and fall times for the VIVE and VIVE Pro are approximately 0.3 and 0.5 ms. The VIVE Pro 2 achieves a very small duty cycle of only 5% with rise and fall times of approximately 0.3 ms by fast switching of the backlight. We also tested the VIVE Pro 2 using the 90-Hz configuration for comparison with the VIVE and VIVE Pro. The temporal characteristics is approximately the same as the VIVE Pro 2 at 120 Hz (e.g., 5% duty cycle at 90 and 120 Hz), with slightly increased pulse width from 0.42 to 0.55 ms.

**Figure 7:**
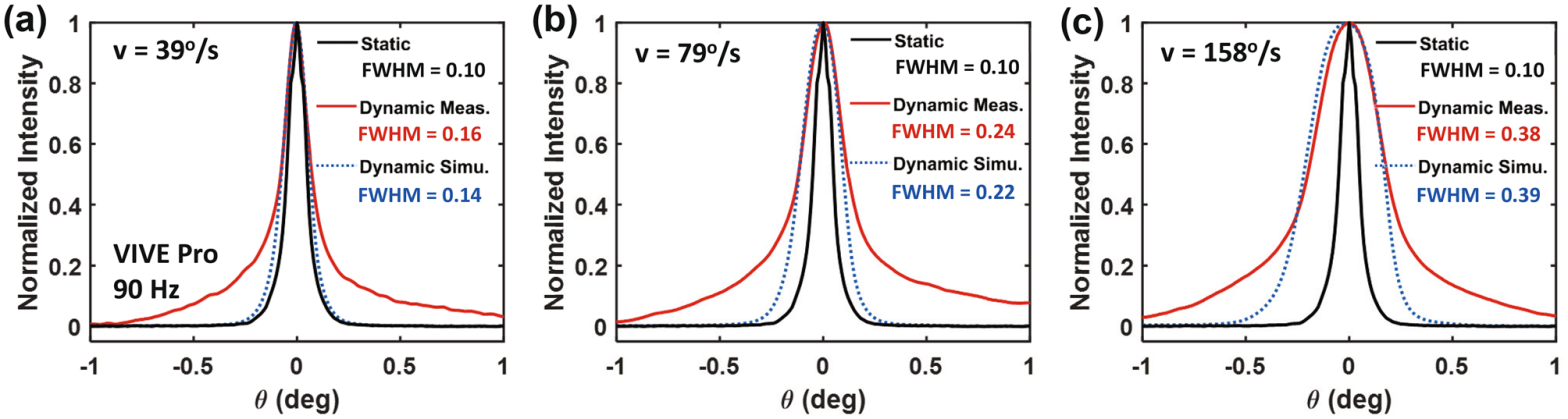
Measured and simulated static and dynamic LSFs for the VIVE Pro at 90 Hz with target motion velocity of 39 (**a**), 79 (**b**) and 158°/s (**c**) during smooth pursuit.

### Spatiotemporal characteristics of VR displays

#### Comparison between measurement and simulation results

We first compare the measured and simulated spatiotemporal responses of the VIVE Pro operating at 90 Hz as measured by the dynamic LSFs shown in Fig. 7, assuming a smooth pursuit eye movement. The dynamic LSFs demonstrate increased full width at half maximum (FWHM) of approximately 0.16, 0.24 and 0.38$^{\circ }$ corresponding to line motion velocity of 39, 79 and 158°/s, compared to the static FWHM of 0.10$^{\circ }$, indicating more substantial motion blur with increased velocity. The slight negative shift of the dynamic LSF at 158°/s is originated from the waveform falling edge during exponential decay (see Eq. 4). The simulations that combine the spatial and temporal characteristics closely match the measurement results with very small absolute errors in dynamic FWHM within 0.02$^{\circ }$, which is only about 36% of the display pixel pitch of the VIVE Pro. The relative discrepancies between the simulation and measurements are about 12.5, 8.3, and 2.6% for motion speeds of 39, 79, and 158°/s, respectively. The relative mismatch reduces by increasing the motion speed indicating accurate model of motion in the spatiotemporal performance.

Although the high-speed camera pco.dimax cs4 achieves a fast frame rate of 1080 Hz (period of 0.93 ms), it is not capable of capturing the spatiotemporal characteristics of the high refresh rate VIVE Pro 2 at 120 Hz with 5% duty cycle (pulse width of 0.42 ms). It requires a camera frame rate of at least 4.8 kHz to capture a minimum of two images per emission cycle of the VIVE Pro 2. Besides, the pco.dimax cs4 camera shows a low-frequency veiling glare that results in long-range tails in the measured dynamic LSFs (see Fig. 7). This may also lead to a slightly overestimated FWHM of 0.02° in the measurements compared to that of the theoretical simulation especially for motion speed of 39°/s. On the other hand, the FLIR camera used in the static spatial measurements is not affected by the veiling glare. Therefore, in the following sections, we use the validated spatiotemporal simulations to characterize the spatiotemporal response of the faster VIVE Pro 2 and to extract the dynamic MTFs to avoid optical contamination by the camera glare.

#### Spatiotemporal resolution

**Figure 8:**
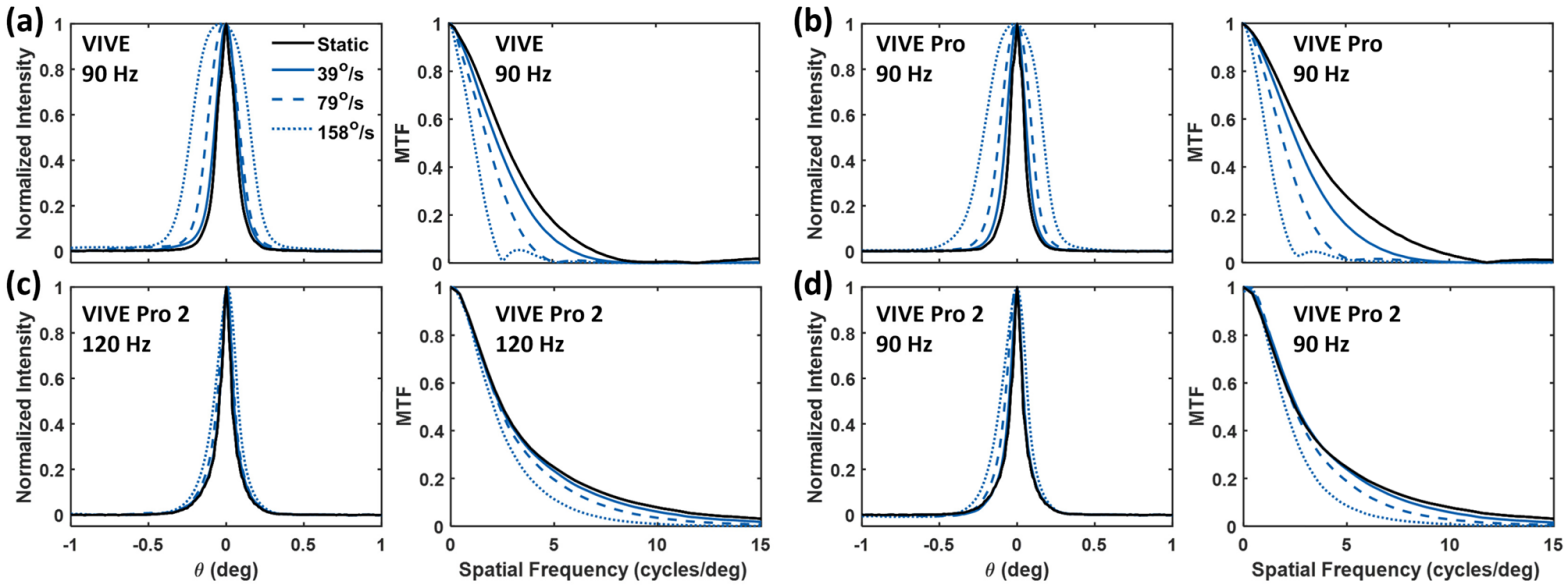
Simulated static and dynamic LSFs and MTFs for (**a**) the VIVE at 90 Hz, (**b**) the VIVE Pro at 90 Hz, (**c**) the VIVE Pro 2 at 120 Hz, and (**d**) the VIVE Pro 2 at 90 Hz during a smooth-pursuit eye movement.

Figure 8 compares the simulated static and dynamic LSFs and MTFs with 39, 79 and 158$^{\circ }$/s motion velocity for the VIVE and VIVE Pro at 90 Hz, as well as VIVE Pro 2 at 120 Hz and 90 Hz. Compared to the VIVE with the same display refresh rate and duty cycle (17%), the VIVE Pro shows improved static and thus dynamic MTF during smooth pursuit of the moving target. For example, the dynamic MTFs show that, compared to the VIVE, the cutoff frequency of the VIVE Pro increases from approximately 7.5 to 10 cycles/degree with a target motion speed of 39°/s.

The VIVE Pro 2 presents the highest static spatial resolution among the three evaluated HMDs with a static FWHM of approximately 0.07$^{\circ }$. At the same time, it increases the refresh rate to 120 Hz while reducing the duty cycle to 5%. As a result, the pulse width of the VIVE Pro 2 is substantially reduced to 0.42 ms compared to approximately 1.9 ms of the VIVE and VIVE Pro. Therefore, the VIVE Pro 2 demonstrates improved spatiotemporal resolution with a dynamic FWHM of approximately 0.1° at a motion speed of 39°/s. It is also indicated in Fig. 8c,d that further increasing the refresh rate from 90 to 120 Hz does not improve the spatiotemporal performance of the VIVE Pro 2 with a very small duty cycle of 5%.

### Impact of display refresh rate and duty cycle

**Figure 9:**
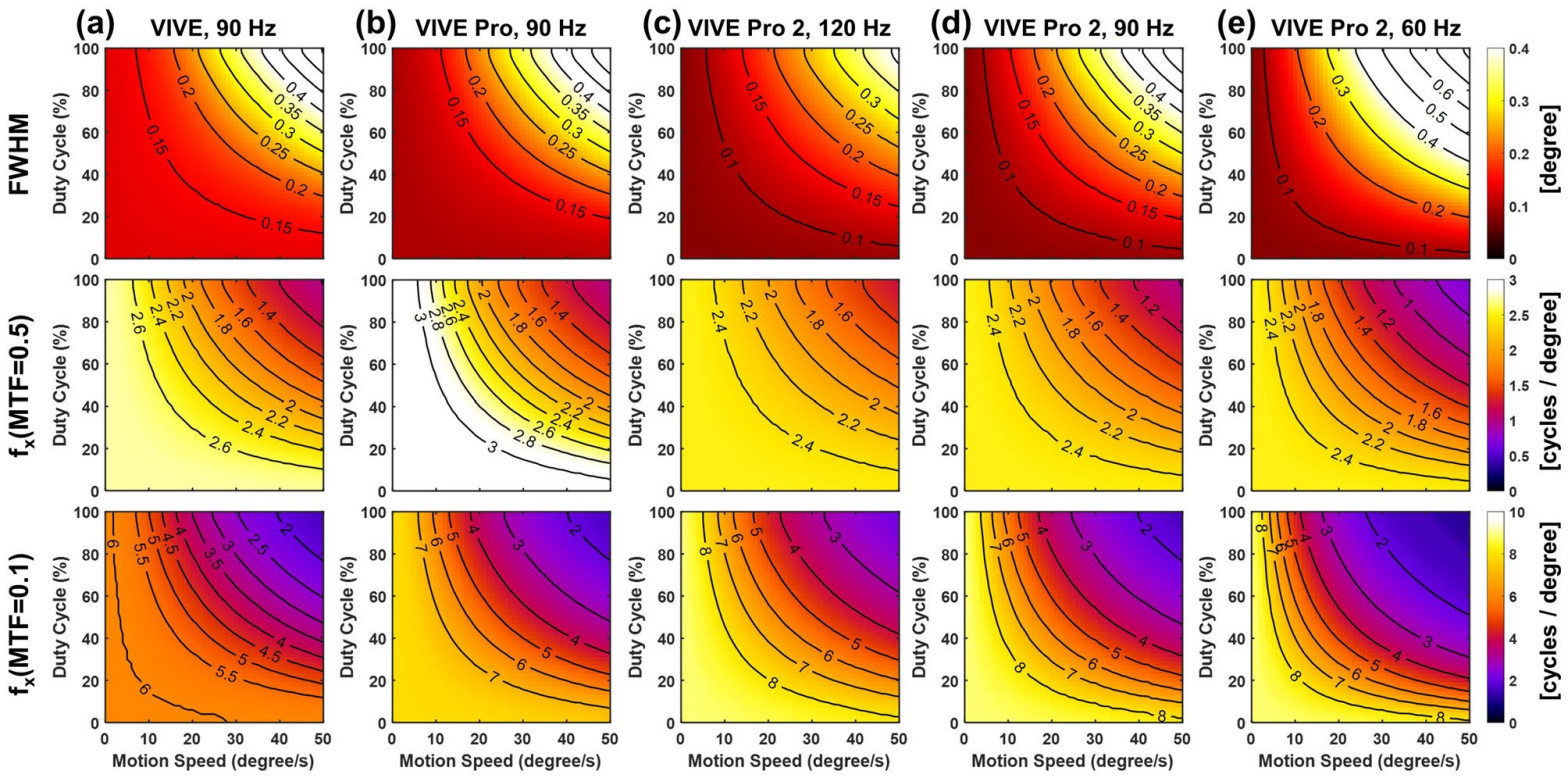
FWHM (first row), spatial frequencies at MTF = 0.5 (second row) and 0.1 (third row) with varied display duty cycle and motion speed for (**a**) the VIVE at 90 Hz, (**b**) the VIVE Pro at 90 Hz, (**c**) the VIVE Pro 2 at 120 Hz, (**d**) the VIVE Pro 2 at 90 Hz, and (**e**) the VIVE Pro 2 at 60 Hz.

The spatiotemporal resolution characteristics indicates that the motion blur can be mitigated by increasing the display refresh rate or reducing the duty cycle of the emission. As shown in Fig. 9, we investigate the impacts of display refresh rate and duty cycle on the spatiotemporal performance of the VR HMDs using different temporal settings. The spatiotemporal resolution is characterized by the FWHM of the dynamic LSFs, and spatial frequencies at dynamic MTFs equal to 0.5 ($f_x(MTF = 0.5)$) and 0.1 ($f_x(MTF = 0.1)$). At each display configuration, we vary the display duty cycle and target motion speed up to 50°/s.

Figure 9a–c compares the spatiotemporal performances of the VIVE, VIVE Pro and VIVE Pro 2 at maximized display refresh rate (90 Hz for the VIVE and VIVE Pro, and 120 Hz for the VIVE Pro 2). It is illustrated that using pulsed emission can substantially reduces the dynamic FWHM. For instance, the dynamic FWHM drops from greater than 0.4° using 100% duty cycle (as in conventional flat-panel displays) to less than 0.15° using 17% duty cycle as in the VIVE and VIVE Pro at 40°/s target motion speed. Regarding the dynamic resolution, the VIVE Pro achieves the best low to mid-frequency spatiotemporal resolution at MTF = 0.5. For instance, $f_x(MTF = 0.5)$ is approximately 3 cycles/$^{\circ }$ for the VIVE Pro at 40°/s motion speed and 17% duty cycle, compared to approximately 2.4 cycles/$^{\circ }$ for the VIVE Pro 2 at the same condition. On the other hand, the VIVE Pro 2 gains resolution in the high frequency range with improved $f_x(MTF = 0.1)$ to approximately 8 cycles/$^{\circ }$ at 40°/s motion speed and 5% display duty cycle, compared the VIVE Pro of approximately 7 cycles/$^{\circ }$. In other words, the VIVE Pro 2 improves the high-frequency performance by increasing pixel resolution, however, the additional image blur by the LCD module of the VIVE Pro 2 may limit its low and mid-frequency range performance.

Figure 9c,d demonstrates that if pulsed emission is used (e.g., with less than 20% duty cycle), increasing the display refresh rate from 90 to 120 Hz does not vary the spatiotemporal performance significantly. However, using a 60 Hz refresh rate in combination with a short single pulse per frame may lead to display flickering and visual disturbances. On the other hand, the enhancement in spatiotemporal resolution is prominent by increasing display refresh rate from 60 to 120 Hz, if continuous emission (100% duty cycle) is used.

### Visualizations and image quality under motion

#### Temporal motion artifacts

Figures 8 and 9 illustrate the dynamic characteristics of VR HMDs, assuming ideal display response (no temporal latency or artifacts) and a perfect smooth pursuit eye tracking. Next, in this section, we expand the evaluation and discussion to include potential temporal image artifacts in several possible user scenarios.

**Figure 10:**
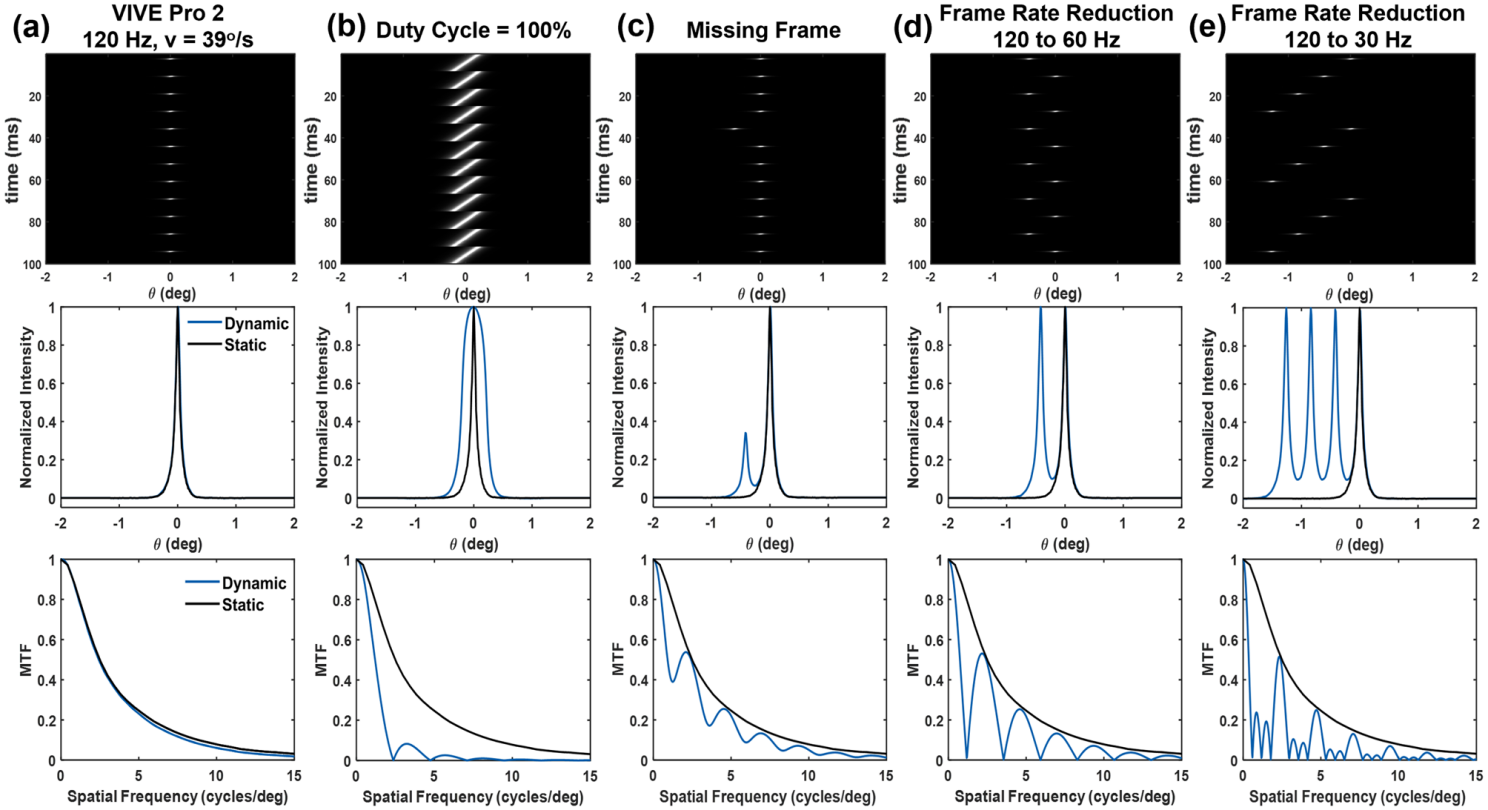
Spatiotemporal illustrations (first row), static and dynamic LSFs (second row) and MTFs (third row) of a moving line at 39°/s motion speed on the VIVE Pro 2 with 120 Hz refresh rate (**a**). Illustrations of motion image artifacts of the line by (**b**) a continuous emission with 100% duty cycle, (**c**) missing a single frame update, and frame rate reduction from 120 Hz to (**d**) 60 Hz and (**e**) 30 Hz.

Pulsed emission during smooth pursuit: As the baseline, Fig. 10a shows the spatial-temporal distribution of the moving line in the VIVE Pro 2 at 120 Hz and 39°/s. The static and dynamic LSFs and MTFs are shown as the reference spatiotemporal performance. This case represents the native spatiotemporal performance of the VIVE Pro 2 HMD at the maximum frame rate, while other user scenarios below assume temporal motion artifacts such as long emission cycle, missing frames and reduced frame rate.

Continuous emission: Figure 10b extends the emission time from 5% duty cycle in (a) to 100% assuming a continuous emission. The emission spans the entire frame. Compared to the baseline, the emission time is about 20 times longer resulting in a much longer translation of about $-v/f$ per frame in smooth pursuit. As a result, the dynamic LSF under continuous emission is substantially broadened with a FWHM of 0.35$^{\circ }$. The cutoff spatial frequency is only approximately 2.5 cycles/$^{\circ }$ indicating motion induced image blur.

Missing frame: Potential latency between motion and display photon emission may result in delay in content update within a single or multiple frames. Figure 10c illustrates an example similar to (a), but the sync of a single frame is missed due to potential latency or a temporary drop in refresh rate. As a result, a low-luminance shadow image is added to the primary visualization, assuming a human eye integration rate of 30 Hz, with a lateral shift of *v*/*f* of 0.33$^{\circ }$.

Frame rate reduction: If the display signal/data is rendered at a reduced refresh rate (e.g., 60 or 30 Hz as shown in Fig. 10d,e) with respect to the display refresh rate of 120 Hz, multiple images can be visualized during smooth pursuit of the moving target. Such a frame rate reduction can be associated with graphic hardware limitations when rendering of heavily loaded content or an original lower refresh rate of moving scene itself (e.g., the headset is used to render a low frame rate video).

**Figure 11:**
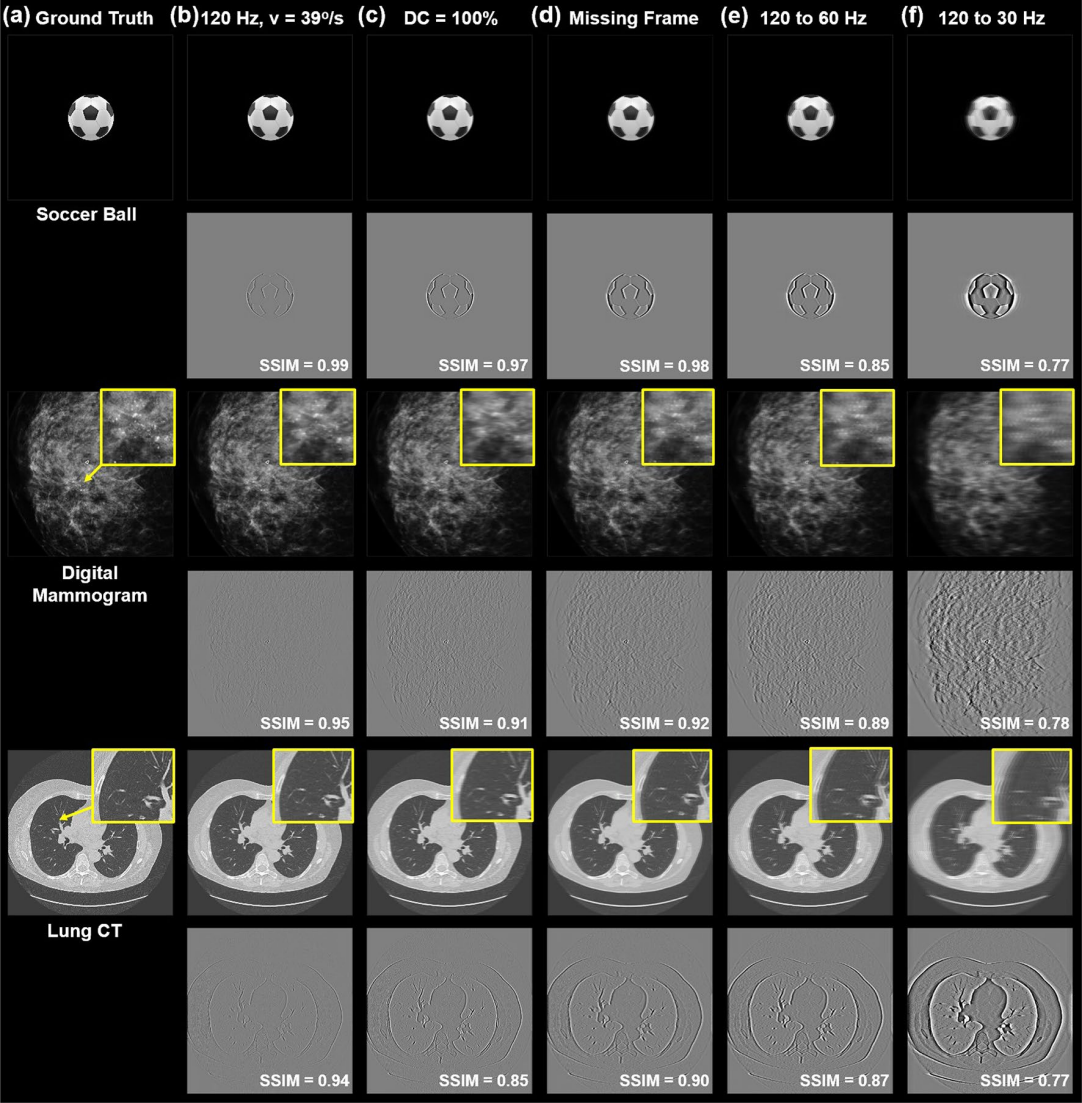
Visualizations of motion image artifacts and motion image errors (difference images with respect to static images) of a soccer ball (first row), a digital mammography image (second row) and a lung CT slice (third row) in motion: (**a**) referencing static images, (**b**) moving scenes at 39°/s using a pulsed 120 Hz refresh rate, (**c**) a continuous emission with 100% duty cycle, moving images with a missing frame (**d**) and frame rate reduction from 120 Hz to 60 Hz (**e**) and 30 Hz (**f**).

#### Image quality under motion

Finally, we simulate the synthesized images during smooth-pursuit eye movement using the dynamic MTF ($MTF_d$) obtained for each scenario with motion artifacts to blur the static images ($I_s(x,y)$). The synthesized dynamic image $I_d(x,y)$ under a specific display configuration can be expressed as the inverse Fourier transform of the dynamic MTF blurred image in the spatial frequency domain:

6 $$\begin{aligned} I_d(x, y) = FFT^{-1}\{FFT[I_s(x,y)]\cdot MTF_d(f_x, f_y)\}. \end{aligned}$$

Figure 11 shows three example visualizations ($I_d(x,y)$) and the difference images of the motion contaminated scenes with respect to the static images (i.e., $I_d(x,y) - I_s(x,y)$) corresponding to motion artifact scenarios in the previous section. We adopt the spatiotemporal performance of the VIVE Pro 2 at 120 Hz and a 39°/s motion speed. The image dimension is 40 $\times$ 40° for all three examples. The first example shows a flying soccer ball assuming the human is tracking the moving target. The second and third examples represent medical image visualizations in different modalities such as digital mammography and lung CT. A realistic scenario could be the medical image is presented in static view, while the user is in motion (e.g., head rotation in search or motion of the user in the 3D space). The error maps in Fig. 11 illustrate the magnitude of motion blur in each scenario. The errors are increased especially with multiple image artifacts in (d–f). The overlaid shadow images increases the motion-induced error by a wide lateral shift of more than 1$^{\circ }$. in (f) when the content frame rate is reduced to 30 Hz.

To quantify the motion contaminated image quality, we compute the structural similarity (SSIM) index^39^ for each example and configuration with respect to the static ground truth, shown as the difference images in Fig. 11. The SSIM measures the local means, standard deviations, and cross-covariance between the motion contaminated and reference static images. As illustrated in Fig. 11b,c, comparing to pulsed emission with a 5% duty cycle, continuous emission increases the image blur and reduces the SSIM of the dynamic visualization (e.g., SSIM reduces from 0.94 to 0.85 for the lung CT image in motion). Missing frame or reduction in frame rate, as shown in Fig. 11d–f results in overlaid double or multiple images that can severely degrade the image quality in motion. For instance, the SSIMs of the soccer ball and digital mammography images reduce from 0.99 to 0.77 and from 0.95 to 0.78 respectively, if the content refresh rate is only 30 Hz. As shown in the magnified regions of interest (ROIs) in Fig. 11, the visibility of high-resolution anatomical details such as the microcalcifications as early indications of breast cancer in the digital mammogram and bronchioles in the lung CT slice are degraded by motion artifacts.

## Discussion

We investigated the spatial, temporal and spatiotemporal characteristics of VR HMDs. A spatiotemporal model was developed based on the measured spatial and temporal characteristics of VR HMDs. The VIVE Pro 2 with an increased pixel resolution demonstrates improved spatial resolution in the high frequency range. However, the low and mid-frequency resolution may suffer from optical blur from the liquid crystal module, compared to the VIVE Pro using the PenTile OLED pixel architecture. In the temporal domain, the VIVE Pro 2 achieves a fast 120 Hz refresh rate with a short 5% duty cycle yielding a display emission time of only 0.42 ms per frame. The fast response of the VIVE Pro 2 reduces motion blur in smooth pursuit eye tracking. Comparing different temporal settings reveals that reducing the duty cycle is very effective in mitigating motion blur when pulsed display emission is used. The emission duty cycle of less than 20% implemented in the evaluated VR HMDs improves the image resolution especially with fast target motion speed greater than 20°/s in smooth pursuit. Although increasing the refresh rate is beneficial for continuous display emission, its reduction in smooth pursuit motion blur is limited if a VR HMD has already implemented pulsed emission with a small duty cycle less than 20%. The visualizations of synthesized images in motion also demonstrates that pulsed emission is recommended for VR HMDs to reduce motion blur. In addition, the content frame rate should match the display refresh rate to avoid substantial motion blur by replicated shadow images.

The findings of this work apply to VR applications in both computer graphics and medical imaging. In computer graphics when there is motion in the scene, e.g., in video games, the temporal response should be optimized to mitigate potential motion blur. On the other hand, for VR applications in medicine, e.g., search for abnormalities in a diagnostic image including mammography or CT, the image content is static in the 3D space, but the observer is searching for a potential target or lesion. In this case, the observer may rotate the head or move in the 3D space creating relative motion of the image with respect to the eyes. If the VR headset is used for displaying a live scene, e.g., live ultrasound, the refresh rate of the content can be limited. This may result in substantial image blur due to overlaid multiple shadow images while the observer is in motion.

The results of this study can be potentially used to guide the hardware design of future VR headsets. Improving VR image quality and user experience demands coordinated advancement of both spatial and temporal performance. For example, when a fast motion (e.g., greater than 10°/s) is involved, the spatiotemporal resolution is dominated by the motion speed and device temporal performance. Therefore, pulsed emission in combination with fast display frame rate matching the input content is desirable to mitigate motion blur and maximize the temporal performance. However, it is challenging to render high quality visual content at ultra-high frame rate due to limited GPU performance. An adaptive refresh rate and resolution technique has been reported to compromise the spatial and temporal resolution using a perceptual visual model given the object motion behavior^26^. On the other hand, if motion is very slow, visual perception is limited by spatial resolution. The HMDs evaluated in this work achieve a static spatial resolution of only about 10 cycles/$^{\circ }$ at the center of the display. However, the human eye contrast sensitivity peaks at 4 cycles/$^{\circ }$ with a perceptual limit up to approximately 60 cycles/$^{\circ }$^40^, indicating that the display pixelation and spatiotemporal image blur are still visible. Thereby, it requires continuous efforts in increasing display resolution and developing efficient rendering methods. Advanced techniques such as gaze-dependent foveated rendering with eye tracking have been extensively investigated to reduce the GPU workload by reducing the spatial image quality in peripheral vision^41–43^.

There are several limitations in the current study. First, location-dependent spatiotemporal performance of VR HMDs has not yet been evaluated. Our previous work has shown that the VR displays suffers from image blur at the periphery of the display FOV. Therefore, spatiotemporal characteristics are not uniform across the display FOV and more severe motion blur is expected at the periphery. In combination with chromatic aberrations^23^, color shift at the edge of an object is also expected during motion. We believe the methodologies and results presented in this work can be extended to evaluate the perceptual performance. VR devices present stereo image pairs and the image quality experienced by the user is dependent on the fusion of images in both eyes. Therefore, the impacts of location-dependent image quality and binocular perception will need to be considered in future work. To assess the spatiotemporal perceptual limits, human and/or model observer studies should be conducted using well-specified spatial and temporal imaging tasks. In addition, this study assumes an ideal smooth-pursuit eye movement with respect to the target, i.e., perfectly matched target and gaze position and eye velocity. The latency of smooth pursuit response at the beginning of the tracking process is ignored. This study only evaluates the smooth pursuit eye tracking with a limited motion speed, without involving saccadic eye movements or fixation. Further, this study only investigates the spatiotemporal performance of VR display hardware. Impact of latency during the sensing, tracking, and rendering processes on the spatiotemporal response is not included in the analysis and is out of the scope of this article. Finally, the spatiotemporal effect on noise has not been evaluated for VR HMDs. This study only involves motion blur in the spatial resolution without correlation of noise in the spatiotemporal domain. The image quality and visibility of a moving target on a noisy background, static or dynamic, remains to be studied in future work.

The outcomes of this study provide both measurement and modeling methods to characterize the spatiotemporal response of numerous VR HMDs on the market. This work helps bridge the regulatory science gaps between VR hardware design and objective image quality when image or user motion exists, for the purpose of ensuring safe and effective use of these devices in medical applications.

## Data Availability

The datasets used and/or analyzed during the current study are available from the corresponding author on reasonable request.
